# Mesonephric-like Adenocarcinoma of the Uterine Corpus: Genomic and Immunohistochemical Profiling with Comprehensive Clinicopathological Analysis of 17 Consecutive Cases from a Single Institution

**DOI:** 10.3390/biomedicines11082269

**Published:** 2023-08-15

**Authors:** Hyun-Hee Koh, Eunhyang Park, Hyun-Soo Kim

**Affiliations:** 1 Department of Pathology, Severance Hospital, Yonsei University College of Medicine, Seoul 03722, Republic of Korea; hhkoh0301@gmail.com; 2 Department of Pathology and Translational Genomics, Samsung Medical Center, Sungkyunkwan University School of Medicine, Seoul 06351, Republic of Korea

**Keywords:** uterus, mesonephric-like adenocarcinoma, targeted sequencing, immunohistochemistry, programmed cell death-ligand 1, mismatch repair

## Abstract

Data on genetic and immunophenotypical characteristics of uterine mesonephric-like adenocarcinoma (MLA) remain limited. Therefore, we aimed to investigate the clinicopathological, immunohistochemical, and molecular features of uterine MLA. We performed targeted sequencing, array comparative genomic hybridization, and immunostaining in 17, 13, and 17 uterine MLA cases, respectively. Nine patients developed lung metastases. Eleven patients experienced disease recurrences. The most frequently mutated gene was Kirsten rat sarcoma viral oncogene homolog (*KRAS*; 13/17). Both the primary and matched metastatic tumors harbored identical *KRAS* (3/4) and phosphatase and tensin homolog deleted on chromosome 10 (1/4) mutations, and did not harbor any additional mutations. A total of 2 of the 17 cases harbored tumor protein 53 (*TP53*) frameshift insertion and deletion, respectively. Chromosomal gains were detected in 1q (13/13), 10 (13/13), 20 (10/13), 2 (9/13), and 12 (6/13). Programmed cell death-ligand 1 overexpression or mismatch repair deficiency was not observed in any of the cases. Initial serosal extension and lung metastasis independently predicted recurrence-free survival with hazard ratios of 6.30 and 7.31, respectively. Our observations consolidated the clinicopathological and molecular characteristics of uterine MLA. Both clinicians and pathologists should consider these features to make an accurate diagnosis of uterine MLA and to ensure appropriate therapeutic management of this rare entity.

## 1. Introduction

Endometrial carcinoma (EC) is the sixth leading cause of carcinoma-related death among women worldwide [1,2,3]. The incidence rates of EC have been steadily increasing, particularly in developed countries [4,5]. The diagnosis of EC subtypes is based on their distinct morphological features [6]. Endometrioid carcinoma accounts for the majority of EC cases, followed by serous carcinoma, clear cell carcinoma, carcinosarcoma, and undifferentiated carcinoma [6]. The histological type and grade as well as the International Federation of Gynecology and Obstetrics (FIGO) staging guides EC prognosis [6,7]; however, histological features overlap significantly between some subtypes, which makes accurate classification difficult. Consequently, continued efforts have been made to develop ancillary techniques, such as immunohistochemical staining (IHC) and molecular analyses, to stratify EC patients.

Mesonephric-like adenocarcinoma (MLA) is a rare but distinct gynecological malignancy, primarily arising in the uterine corpus and adnexa. MLA was recently introduced as a new histological type in the 2020 version of the World Health Organization Classification of Female Genital Tumors [6]. Uterine MLA exhibits morphological, immunophenotypical, and genetic characteristics similar to those of mesonephric adenocarcinoma (MA), which is a rare malignant tumor derived from the mesonephric remnants located in the lateral wall of the vagina and uterine cervix [8]. The term MLA has been used for malignant mesonephric lesions arising in the uterine corpus [3,9,10], because it still debated whether they are of mesonephric origin. Both clinicians and pathologists should be aware of the clinical, pathological, and molecular features of uterine MLA to make an accurate diagnosis and to ensure appropriate therapeutic management.

Next-generation sequencing (NGS), also known as massively parallel sequencing, allows for the effective capture of a substantial amount of genomic information regarding tumor development, progression, and biological behavior [11]. NGS is inextricably intertwined with the realization of precision medicine in oncology. While it is unlikely to obviate traditional pathological diagnosis in its current state, NGS allows a more complete picture of carcinogenesis, progression, and metastasis than can be seen with any other modality. Recent advances in sequencing technologies have provided substantial insights into the mutated carcinoma-related genes and mutational processes operative in EC [12,13]. Novel classification systems that incorporate molecular features have been developed to provide objective and reproducible EC categorization [14]. The addition of molecular and immunohistochemical markers allows for the identification of clinically relevant subgroups, with potential therapeutic implications [15]. However, despite significant advances directed toward elucidating molecular mechanisms and developing clinical trials for patients with EC, data on specific genetic alterations and molecular characteristics of uterine MLA remain limited [3,16,17].

Immune checkpoints and inhibitory immunoreceptors, including programmed cell death protein 1 (PD-1) and its ligand (PD-L1), have gained attention as therapeutic targets [18]. PD-L1 expression on tumor and immune cells can be detected using IHC and different PD-L1 commercial clones. PD-1 and PD-L1 play progressively important roles in our understanding of tumor immunology and antitumor treatment [19]. Binding of PD-L1 to its receptor PD-1 leads to T-cell inactivation in a variety of carcinomas [20]. Therefore, anti-PD-1/PD-L1 treatment deregulates the adverse impact of tumor-infiltrating T-cells, which in turn may reverse the tumor immune resistance [19]. Several clinical and experimental studies have investigated PD-L1 expression in EC and its prognostic values as well as its efficacy as an immunotherapy for EC [21,22,23]; however, these studies focused on endometrioid carcinomas and did not consider MLA as a separate or independent group. In addition, clinical trials that investigated the anti-PD-1 antibody, pembrolizumab, as a treatment for advanced or recurrent EC [24] did not separate MLA from other histological EC types.

Microsatellite instability (MSI), caused by mismatch repair deficiency (MMRd), induces high numbers of mismatches, insertions, and deletions. MSI/MMRd identification in EC is clinically important because it may guide adjuvant therapy and improve prognosis [25,26,27]. MSI/MMRd tumors are more immunogenic; therefore, they are more responsive to immune checkpoint inhibitors [28,29]. When screening MMRd cases, IHC is the standard for detecting MMR protein expression [30,31,32]. In addition, polymerase chain reaction (PCR)-based MSI testing is also used; however, less frequently.

Our group has steadily documented the clinical manifestations, cytological and histological features, immunophenotypes, and mutational profiles of uterine MLA using IHC and molecular analyses [2,3,33,34,35,36,37,38,39,40]. However, get a deep insight into their relevance, the results need to be consolidated and completely analyzed. Therefore, in this study, we comprehensively investigated the clinicopathological, immunohistochemical, and molecular characteristics of uterine MLA and determined their relationships and prognostic significance.

## 2. Materials and Methods

### 2.1. Case Selection and Clinicopathological Data Collection

**Figure 1:**
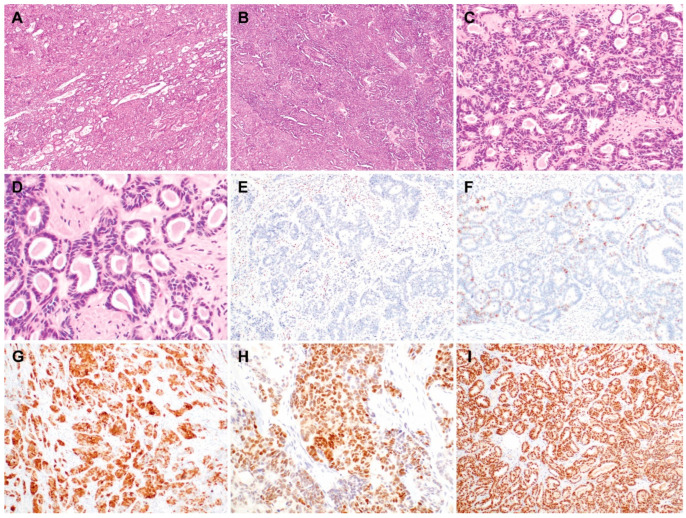
Histological and immunophenotypical features of uterine mesonephric-like adenocarcinoma. (**A**) Tubular and glandular patterns showing compactly aggregated small-to-medium-sized tubules and elongated ductal structures. (**B**) Solid and tubular patterns showing solid cellular sheets and slit-like tubular lumina. (**C**) Complex small tubular proliferation with back-to-back arrangement. (**D**) Eosinophilic, hyaline- or colloid-like intraluminal secretions. (**E**) Lack of estrogen receptor expression. (**F**) Wild-type p53 immunostaining pattern. (**G**) Non-diffuse p16 positivity. (**H**) Moderate-to-strong nuclear immunoreactivity for transcription termination factor 1. (**I**) Uniform and strong GATA-binding protein 3 expression.

**Figure 2:**
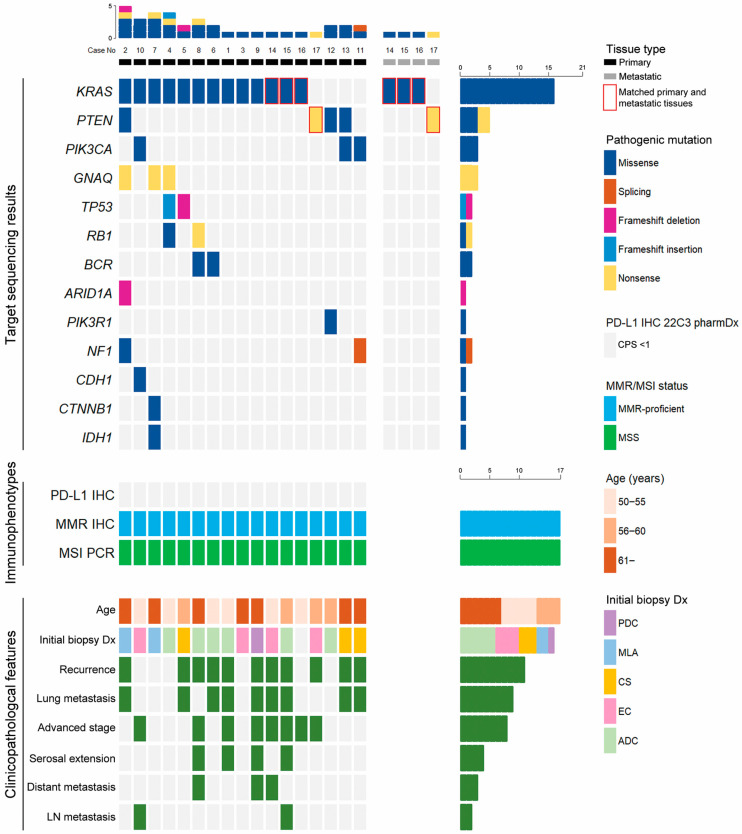
Illustrations of targeted sequencing and immunohistochemical staining (IHC) displaying programmed cell death-ligand 1 (PD-L1) expression, presence of mismatch repair (MMR) proteins, microsatellite instability (MSI) status, and clinicopathological features of uterine mesonephric-like adenocarcinoma (MLA). Most of the MLA cases harbor Kirsten rat sarcoma viral oncogene homolog (*KRAS*) mutations. Frameshift mutations of tumor protein 53 (*TP53*) are detected in two cases of uterine MLA. None of the examined cases show PD-L1 overexpression. All MLAs are MMR-proficient, microsatellite-stable tumors. During initial biopsy, only 2 of the 17 patients are correctly diagnosed as MLA, whereas the remaining 15 patients are misdiagnosed as endometrioid carcinoma (EC) or carcinosarcoma (CS). Both post-operative recurrences and lung metastases are identified in more than half of uterine MLA patients. ADC—adenocarcinoma; *ARID1A*—AT-rich interactive domain 1A; *BCR*—breakpoint cluster region; *CDH1*—cadherin 1; CPS—combined proportion score; *CTNNB1*—catenin beta 1; Dx—diagnosis; *GNAQ*—guanine nucleotide binding protein subunit alpha Q; *IDH1*—isocitrate dehydrogenase 1; LN—lymph node; MSS—microsatellite-stable; *NF1*—neurofibromin 1; PCR—polymerase chain reaction; PDC—poorly differentiated carcinoma; *PIK3CA*—phosphatidylinositol-4,5-bisphosphate 3-kinase catalytic subunit alpha; *PIK3R1*—phosphoinositide-3-kinase regulatory subunit 1; *PTEN*—phosphatase and tensin homolog deleted on chromosome 10; *RB1*—retinoblastoma 1.

**Figure 3:**
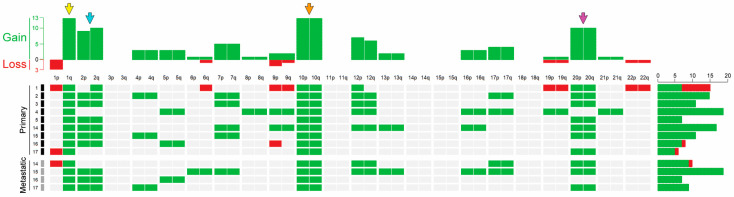
Diagram depicting chromosomal copy number variations, determined using array comparative genomic hybridization. Copy number variations are indicated in green or red for gain or loss in copy number, respectively. All cases of uterine mesonephric-like adenocarcinoma exhibit gains of chromosome 1q (yellow arrow) and 10 (orange arrow), and most of the cases exhibit gains of chromosome 2 (blue arrow) and 20 (purple arrow).

**Figure 4:**
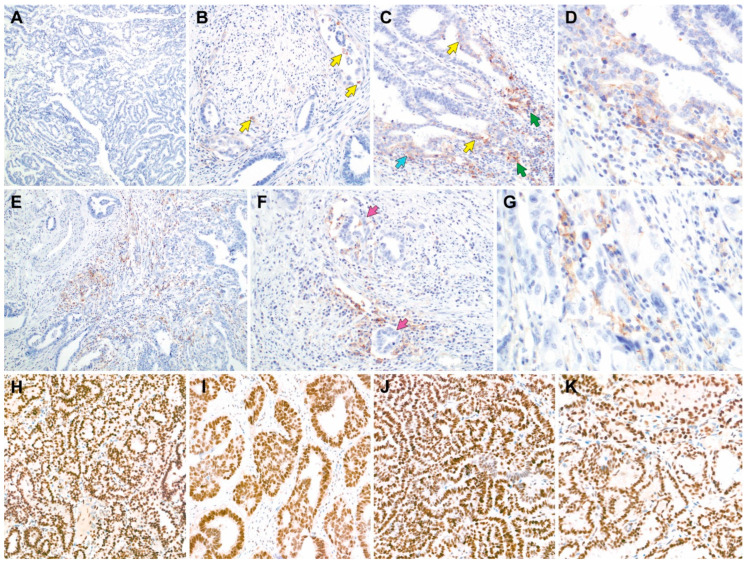
Representative photomicrographs of in uterine mesonephric-like adenocarcinoma programmed cell death-ligand 1 (PD-L1; (**A**–**G**)) and mismatch repair protein (**H**–**K**) immunostaining. (**A**) Case 5: combined proportion score (CPS) 0. (**B**) Case 15: CPS 0.1. Only a few tumor cells (yellow arrows) are positive for PD-L1 with weak-to-moderate staining intensity. (**C**) Case 11: CPS 0.5. Some tumor cells (yellow and blue arrows) and inflammatory cells (green arrows) react with PD-L1 with variable staining intensity. (**D**) Case 11: CPS 0.5. high-power magnification of the left lower corner of image (**C**). Some tumor cells show weak PD-L1 immunoreactivity with membranous and a paranuclear dot-like or Golgi staining pattern. (**E**) Case 12: CPS 0.5. Stromal lymphocytes and plasma cells express PD-L1. (**F**) Case 12: CPS 0.5. The neoplastic glands (purple arrows) are negative for PD-L1, whereas the surrounding inflammatory cells are positive for PD-L1. (**H**) Preserved MutL homolog 1 expression. (**I**) Preserved MutS homolog 2 expression. (**J**) Preserved MutS homolog 6 expression. (**K**) Preserved postmeiotic segregation increased, *Saccharomyces cerevisiae* 2 expression.

**Figure 5:**
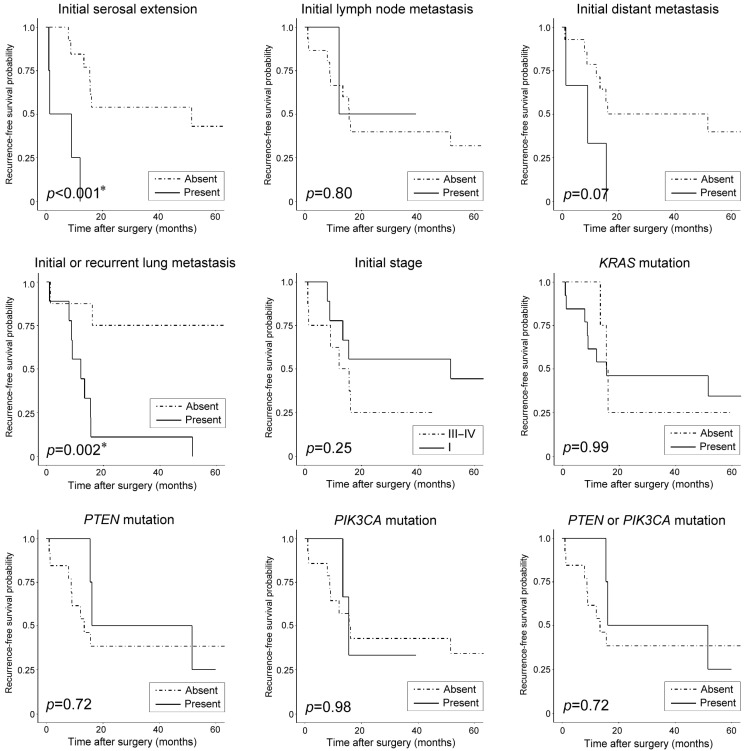
Kaplan–Meier plots showing probability of recurrence-free survival (RFS) stratified by clinicopathological characteristics and pathogenic mutations. Initial serosal extension and lung metastasis significantly predict worse RFS of patients with uterine MLA. *KRAS*—Kirsten rat sarcoma viral oncogene homolog; *PIK3CA*—phosphatidylinositol-4,5-bisphosphate 3-kinase catalytic subunit alpha; *PTEN*—phosphatase and tensin homolog deleted on chromosome 10. * Statistically significant.

**Figure 6:**
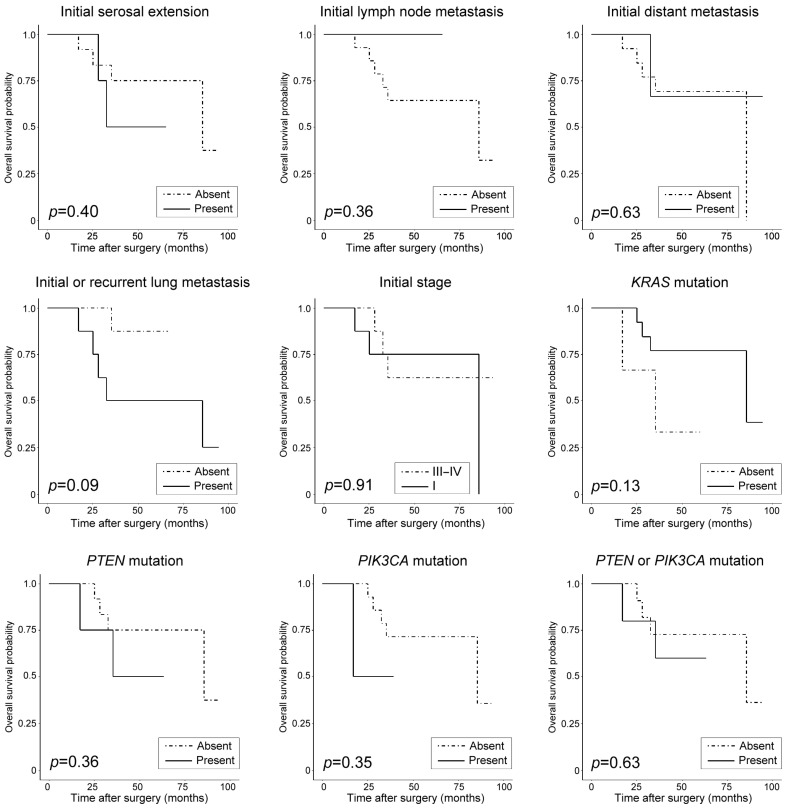
Kaplan–Meier plots showing probability of overall survival (OS) stratified by clinicopathological characteristics and pathogenic mutations. None of the examined parameters significantly predict worse OS of patients with uterine MLA. *KRAS*—Kirsten rat sarcoma viral oncogene homolog; *PIK3CA*—phosphatidylinositol-4,5-bisphosphate 3-kinase catalytic subunit alpha; *PTEN*—phosphatase and tensin homolog deleted on chromosome 10.

We collected 17 cases of uterine MLA from the institutional databases. Two board-certified pathologists reviewed all the available slides to confirm the diagnosis of primary uterine MLA, based on the following morphological, immunophenotypical, and molecular characteristics: (1) architectural diversity (Figure 1A,B); (2) a compact proliferation of small-to-medium-sized tubules lined by cuboidal-to-low columnar epithelia (Figure 1C); (3) eosinophilic, hyaline- or colloid-like secretions noted within the tubular lumina and dilated duct-like structures (Figure 1D); (4) negative or focally positive expression for hormone receptors (Figure 1E); (5) wild-type p53 immunostaining pattern (Figure 1F); (6) non-diffuse p16 positivity (Figure 1G); (7) either at least focally positive expression for one or more mesonephric markers, including transcription termination factor 1 (Figure 1H), GATA-binding protein 3 (Figure 1I), cluster of differentiation 10, or the identification of pathogenic Kirsten rat sarcoma viral oncogene homolog (*KRAS*) mutation, as previously described [2,3,8,9,10,34,36,37,39,40,41,42,43,44]. We reviewed the electronic medical records and pathology reports to collect the following clinical information: age, initial biopsy diagnosis, surgical procedure, hysterectomy diagnosis, uterine serosal extension, lymph node metastasis, distant metastasis, initial FIGO stage, post-operative treatment and recurrence, initial or recurrent lung metastasis, recurrence-free survival (RFS), survival status, and overall survival (OS).

### 2.2. NGS

We used Axen Cancer Panel 2 (Macrogen, Seoul, Republic of Korea) to perform targeted sequencing. We chose the most representative slide, and formalin-fixed, paraffin-embedded (FFPE) tissues were microdissected manually on unstained sections under the microscope. The tumor purity ranged between 75% and 95%. Genomic DNA was extracted using the QIAamp DNA FFPE Tissue Kit (Qiagen, Hilden, Germany). Coding exons and the flanking regions of 171 genes were enriched using the extracted genomic DNA as the template and the SureSelectXT Reagent Kit (Agilent Technologies, Santa Clara, CA, USA). The products were sequenced on a HiSeq 2500 Sequencing System (Illumina, San Diego, CA, USA). We used the Burrows–Wheeler Alignment-maximum exact match (https://bio-bwa.sourceforge.net/; accessed on 10 August 2023) to align the reads to a reference genome sequence (Genome Reference Consortium Human Build 37), obtained from the University of California Santa Cruz Genome Browser database (https://genome.ucsc.edu/; accessed on 10 August 2023). We removed adapter sequences from the raw sequencing reads using Cutadapt (https://cutadapt.readthedocs.io/; accessed on 10 August 2023). Poorly mapped reads were removed using SAMtools (https://samtools.sourceforge.net/; accessed on 10 August 2023). We identified single nucleotide variants (SNVs), insertions and deletions (indels) using MuTect2 (https://gatk.broadinstitute.org/hc/en-us; accessed on 10 August 2023). Finally, all of the detected variants were annotated using SnpEff and SnpSift (http://pcingola.github.io/SnpEff/; accessed on 10 August 2023) as well as dbNSFP (http://database.liulab.science/dbNSFP; accessed on 10 August 2023). Since this study did not include the matched germline samples, variants satisfying the following criteria were excluded (from the results to reduce the proportion of false-positive or possible germline variants): (1) variants with <5% allele frequency and <100× read depth; (2) variants with an allele frequency >1% in the Genome Aggregation Database (formerly known as the Exome Aggregation Consortium; https://gnomad.broadinstitute.org/; accessed on 10 August 2023); (3) all synonymous, intronic, 30- and 50-untranslated region variants; and (4) variants previously reported to be benign or likely benign with ascertained criteria in the ClinVar (https://www.ncbi.nlm.nih.gov/clinvar/; accessed on 10 August 2023). Finally, pathogenic mutations, described in the Catalogue Of Somatic Mutations In Cancer (https://cancer.sanger.ac.uk/cosmic; accessed on 10 August 2023), and truncating mutations of tumor suppressor genes were selected in analysis. Copy number variation (CNV) log2 ratios were generated using a depth of coverage normalized that of normal uterine tissues.

### 2.3. Array Comparative Genomic Hybridization (CGH)

For each experiment, purified FFPE DNA (500 ng) and reference DNA (500 ng) (NA10851; Coriell Institute for Medical Research, Camden, NJ, USA) were differentially labeled with cyanine 5 (Cy5) and cyanine 3 (Cy3) fluorescent dyes using Universal Linkage System technology (Agilent Technologies), a non-enzymatic direct labeling method. Unreacted dye was removed using KREApure purification columns (Agilent Technologies). DNA labeling efficiency was assessed by NanoDrop 2000 spectrophotometry (ND-2000; Thermo Fisher Scientific, Waltham, MA, USA). The degree of labeling (DoL, the number of fluorophore molecules per 100 nucleotides) was calculated using post-labeling DNA yield and fluorophore concentration. DoL values ranging from 0.75% to 2.5% and 1.75% to 3.5% were considered optimal for Cy5- and Cy3-labeled DNA, respectively. Cy5- and Cy3-labeled DNA hybridization was performed using dual-color array containing 60-mer oligonucleotide probes (SurePrint G3 Human CGH Microarray 8 × 60 K; Agilent Technologies). Following hybridization, the arrays were scanned using a Dual Laser Microarray Scanner (G2565CA; Agilent Technologies). The images were extracted and analyzed using Feature Extraction software version 10.5.1.1 (Agilent Technologies) and DNA Analytics software version 4.0.73 (Agilent Technologies).

### 2.4. MSI Testing

MSI status was determined by multiplex PCR amplification of five quasi-monomorphic mononucleotide repeat markers (BAT-25, BAT-26, NR-21, NR-24, and NR-27) [38,45]. Genomic DNA was isolated from the FFPE sections using a QIAamp DNA Mini Kit (Qiagen). Sense primers were labeled with fluorescent dyes. Amplicons were analyzed on an ABI PRISM 3130 Genetic Analyzer (Applied Biosystems, Foster City, CA, USA). Allelic size was estimated using GeneMapper Software v4.1 (Applied Biosystems), and tumors with allelic size variation in 0, 1, and ≥2 microsatellites were classified as MSS, MSI-low (MSI-L), and MSH-high (MSI-H), respectively.

### 2.5. PD-L1 22C3 Pharmdx IHC

FFPE tissue blocks were cut into 4 μm sections which were subsequently mounted on Superfrost Plus Microscope Slides (Thermo Fisher) dried at 60 °C for 1 hour. PD-L1 IHC was carried out on a Dako Autostainer Link 48 system (Agilent Technologies) using a Dako PD-L1 IHC 22C3 pharmDx kit (Agilent Technologies), with EnVision FLEX visualization system [46]. PD-L1 protein expression was assessed using the combined proportion score (CPS). CPS was calculated as the number of PD-L1-stained cells (tumor cells, lymphocytes, and macrophages) divided by the total number of tumor cells, multiplied by 100. The specimen was considered to have positive PD-L1 expression if CPS ≥ 1 [35,42,46].

### 2.6. MMR IHC

IHC was performed using the Bond Polymer Intense Detection System (Leica Biosystems, Buffalo Grove, IL, USA). Briefly, the 4 μm thick sections cut from FFPE tissue blocks were deparaffinized in xylene and rehydrated through a series of graded alcohols. After antigen retrieval, endogenous peroxidases were quenched with hydrogen peroxide. Next, the sections were incubated with primary antibodies against the MMR proteins MutL homolog 1 (MLH1; prediluted, clone M1, Ventana Medical Systems, Roche, Oro Valley, AZ, USA), MutS homolog 2 (MSH2; dilution 1:500, clone G219-1129, Cell Marque), MutS homolog 6 (MSH6; dilution 1:500, clone 44/MSH6, BD Biosciences, Franklin Lakes, NJ, USA), and postmeiotic segregation increased, *Saccharomyces cerevisiae* 2 (PMS2; dilution 1:20, clone MRQ-28, Cell Marque). A biotin-free polymeric horseradish peroxidase-linker antibody conjugate system was used with a Bond-max automated immunostainer (Leica Biosystems, Buffalo Grove, IL, USA). After chromogenic visualization using 3,3′-diaminobenzidine, the sections were counterstained with hematoxylin, dehydrated in graded alcohols and xylene, and then embedded in a mounting solution. Appropriate controls were stained concurrently. MMRp high-grade serous carcinoma of the ovary served as the positive control. Negative controls were prepared by substituting non-immune serum for primary antibodies, which resulted in undetectable staining.

### 2.7. IHC Interpretation

PD-L1 immunoreactivity in uterine MLA was assessed using the CPS interpretation guideline, as previously described [47]. CPS was calculated as the number of PD-L1-stained cells (viable tumor cells, lymphocytes, and macrophages) divided by the total number of viable tumor cells, multiplied by 100. A minimum of 100 viable tumor cells was considered adequate for evaluating PD-L1 positivity. For tumor cells, partial or complete membranous staining at any intensity was regarded as a positive expression. Membranous and/or cytoplasmic staining at any intensity was regarded as positive for tumor-associated immune cells. MMR protein expression in uterine MLA was classified into three categories: preserved, loss, and subclonal loss [41,48,49]. A lack of one or more MMR protein expression was defined as MMRd, and preserved expression of all four MMR proteins was defined as MMRp. We regarded the complete absence of nuclear staining (0%), in the tumor cells with appropriate internal control staining (positive nuclear expression in the stromal non-neoplastic cells or lymphocytes), as loss of expression [32].

### 2.8. Statistical Analysis

Pearson’s chi-squared test, Fisher’s exact test, or linear-by-linear association test was used to determine the association between recurrent pathogenic mutations and clinicopathological characteristics of patients with uterine MLA. Univariate survival analysis, with the log-rank test and Kaplan–Meier plots (RFS and OS), was conducted to evaluate the prognostic significance of recurrent pathogenic mutations and clinicopathological characteristics. Multivariate survival analysis was performed using the Cox proportional hazards model (95% confidence interval) with the backward stepwise elimination method. All statistical analyses were performed using IBM SPSS Statistics for Windows, version 23.0 (IBM Corporation, Armonk, NY, USA). Statistical significance was defined as *p* < 0.05.

## 3. Results

### 3.1. Clinicopathological Characteristics

Table 1 summarizes the clinicopathological characteristics of 17 patients with uterine MLA. During the initial biopsy, only two patients were correctly diagnosed with MLA. Four patients were misdiagnosed with endometrioid carcinoma. In seven patients whose biopsy specimens were interpreted as adenocarcinoma (6/7) or poorly differentiated carcinoma (1/7), the histological type could not be determined. All patients (17/17) underwent either total (13/17) or radical (4/17) hysterectomy with bilateral salpingo-oophorectomy. Fourteen cases were correctly diagnosed as MLA or mesonephric-like carcinosarcoma; however, three patients were misdiagnosed with endometrioid carcinoma even after hysterectomy. Simultaneously, pelvic lymph node dissection (12/17) and para-aortic lymph node dissection (9/17) or sampling (3/17) was conducted. Four patients had uterine serosal extension, and three had lymph node metastases. The initial FIGO stages were distributed as follows: IA (3/17), IB (6/17), IIIB (3/17), IIIC (2/17), and IVB (3/17). More than two-thirds of patients (12/17) received post-operative treatment, including concurrent chemoradiation therapy (7/12), chemotherapy (2/12), or radiation therapy (3/12). Five of the nine patients with FIGO stage I tumors did not receive any adjuvant treatment. Three of these patients developed recurrent tumors. Overall, 11 patients experienced recurrent disease, primarily in the lung (9/11). Six patients died from the disease. RFS and OS ranged from 1.1 to 66.5 months (median = 15.7 months) and 14.5 to 94.6 months (median = 53.8 months), respectively.

### 3.2. NGS Results

Twenty-one tissue samples obtained from 17 primary and four metastatic tumors were available for SNV analysis, while 13 cases were available for CNV interpretation (Table 2 and Figure 2). Thirteen primary and three metastatic tumors harbored *KRAS* mutations: c.35G>A (6/16), c.35G>T (5/16), c.34G>T (3/16), c.53G>C (1/16), and c.37G>T (1/16). In order of frequency, five mutations (c.804C>A, 2/5; c.892C>T, 2/5; c.70G>C, 1/5) in phosphatase and tensin homolog deleted on chromosome 10 (*PTEN*), three (c.317G>T, 1/3; c.1633G>A, 1/3; c.2740G>A, 1/3) in phosphatidylinositol-4,5-bisphosphate 3-kinase catalytic subunit alpha (*PIK3CA*), three (c.303C>A) in guanine nucleotide binding protein subunit alpha Q (*GNAQ*), as well as two tumor protein 53 (*TP53*) frameshift indels (c.210_211insG, 1/2; c.216delC, 1/2) were detected. Other pathogenic mutations were identified in the breakpoint cluster region (*BCR*; 2/21), neurofibromin 1 (*NF1*; 2/21), retinoblastoma 1 (*RB1*; 2/21), phosphoinositide-3-kinase regulatory subunit 1 (*PIK3R1*; 1/21), AT-rich interactive domain 1A (*ARID1A*; 1/21), catenin beta 1 (*CTNNB1*; 1/21), cadherin 1 (*CDH1*; 1/21), and isocitrate dehydrogenase 1 (*IDH1*; 1/21). Both primary and matched metastatic tumors were analyzed in four cases, in which the same *KRAS* (3/4) or *PTEN* (1/4) mutations were detected. Regarding CNV, frequently amplified genes included neurotrophic receptor tyrosine kinase 1 (*NTRK1*; 10/13), discoidin domain receptor tyrosine kinase 2 (*DDR2*; 8/13), fibroblast growth factor receptor 2 (*FGFR2*; 8/13), anaplastic lymphoma receptor tyrosine kinase (*ALK*: 5/13), erb-B2 receptor tyrosine kinase 4 (*ERBB4*; 4/13), and DNA methyltransferase 3 alpha (*DNMT3A*; 4/13). Three cases exhibited CN loss of Janus kinase 1 (*JAK1*), *JAK2*, *CTNNB1*, *PIK3CA*, and repressor of silencing 1 (*ROS1*).

### 3.3. CGH Results

Thirteen tissue samples obtained from nine primary and four metastatic tumors were available for array CGH analysis. The generated CN plots obtained are shown in Figure 3. The most common alterations observed in MLA were the gains of chromosomes 1q (13/13), 10 (13/13), 20 (10/13), 2 (9/13), and 12 (6/13). These findings were consistent with those of targeted sequencing, demonstrating CN gains in *NTRK1*, *DDR2*, mouse double minute 2 proto-oncogene (*MDM4*), and Abelson murine leukemia viral oncogene homolog 2 (*ABL2*) located in 1q; *FGFR2* and rearranged during transfection (*RET*) in 10q; v-src avian sarcoma (Schmidt–Ruppin A-2) viral oncogene homolog (*SRC*) and guanine nucleotide binding protein, alpha stimulating activity polypeptide 1 (*GNAS*) in 20q; *ALK* and *DNMT3A* in 2p; and *ERBB4* in 2q. CN losses were identified in 1p (3/13), 9p (2/13), 6q (1/13), 9q (1/13), chromosome 19 (1/13), and chromosome 22 (1/13).

### 3.4. PD-L1 Expression, MMR Protein Expression, MSI Status, and Tumor Mutational Burden

Table 3 summarizes the results of PD-L1 expression by IHC and MMR protein detection by multiplex PCR for MSI status determination. Representative photomicrographs, illustrating immunostaining, are depicted in Figure 4. In the majority of cases (14/17), PD-L1 in tumor and tumor-associated inflammatory cells (CPS 0) was not expressed. In the three cases that did express PD-L1, the PD-L1 CPS values were 0.5 (2/3) and 0.1 (1/3). In one of the cases with PD-L1 CPS 0.5, some tumor and inflammatory cells expressed PD-L1 with variable staining intensity. On high-power view, the cells exhibited weak membranous PD-L1 immunoreactivity as well as a paranuclear dot-like and Golgi staining pattern. In the other case with CPS 0.5, stromal lymphocytes and plasma cells expressed PD-L1. While the neoplastic glands were negative for PD-L1, the inflammatory cells surrounding the neoplastic glands and clusters of tumor cells reacted with PD-L1. In the case with PD-L1 CPS 0.1, only a small number of tumor cells expressed PD-L1 with weak-to-moderate staining intensity. Regarding MMR protein expression, all cases (17/17) retained MMR protein staining (for all four MMR proteins), indicating MMRp. Consistent with this finding, 15 cases tested for MSI were interpreted as MSS. Tumor mutational burden was measured using NGS (Table 3). We observed low tumor mutational burden in all examined cases, with a megabase range of 2.70 to 4.72 (median = 3.57).

### 3.5. Clinicopathological and Prognostic Significance of Recurrent Pathogenic Mutations

Based on the pathogenic mutational status, there were no significant differences in clinicopathological characteristics (Table 4) and patient outcomes (Table 5). Univariate survival analysis revealed that initial serosal extension (*p* < 0.001) and initial or recurrent lung metastasis (*p* = 0.002) were significant predictors for RFS. Using multivariate survival analysis, we found that serosal extension and initial or recurrent lung metastasis were independent factors for RFS prediction, with hazard ratios of 6.30 (*p* = 0.037) and 7.31 (*p* = 0.02), respectively. None of the clinicopathological characteristics and recurrent pathogenic mutations were significantly associated with OS. Figure 5 and Figure 6 display Kaplan–Meier plots, with RFS and OS stratified by clinicopathological characteristics and pathogenic mutations, respectively.

## 4. Discussions

In this study, we investigated the genetic features of 17 uterine MLA cases from a single institution. Seventeen primary and four matched metastatic tumors were available for SNV analysis. The most frequently mutated gene was *KRAS*, followed by *PTEN*, *PIK3CA*, *GNAQ*, *CTNNB1*, and *ARID1A*. The obtained results were consistent with that of previous data [3,40,50,51,52], where *KRAS* mutations were found in the majority of uterine MLA cases (16/21). In recent studies, we demonstrated that all examined uterine (6/6) and ovarian (4/4) MLA cases harbored pathogenic *KRAS* mutations [3,10]. In addition, we summarized previously reported genetic abnormalities associated with ovarian MLA [10] and found that the most frequently mutated gene was *KRAS* (23/28), while *PIK3CA*, *NRAS*, and *ARID1A* mutations were uncommon. da Silva et al. [52] reported that the majority of the ovarian and uterine MLAs harbored mutations in *KRAS* (25/28) and genes frequently mutated in Mullerian tumors, including *PIK3CA*, *CTNNB1*, and *PTEN*. Collectively, the data support the concept found by Kolin et al. [51], which states that *KRAS* mutations can be considered as one of the defining features of MLA. Although *KRAS* mutations are not unique to MLA [3,10,39,40,53] and can be identified in 20–30% of the endometrial endometrioid carcinoma [54,55,56], with the additional use of histological features and immunophenotypes characteristic of MLA, the identification of *KRAS* mutations can confirm MLA diagnosis.

We demonstrated that both the primary and matched metastatic tumors harbored identical *KRAS* (3/4) and *PTEN* (1/4) mutations, and did not harbor any additional mutations. da Silva et al. [52] analyzed the mutational profiles of two primary uterine MLAs and their respective distant metastases. Consistent with our result, in one case, both the primary and metastatic tumors harbored an identical *KRAS* mutation, without additional mutations. However, in the other case, the metastatic tumor exhibited identical *KRAS* mutations to the primary tumor and harbored additional mutations in genes related to the mitogen-activated protein kinase pathway (mitogen-activated protein kinase kinase kinase 13, mesenchymal epithelial transition, and mitogen-activated protein kinase 3). The latter finding suggests that the progression from primary to metastatic MLA may involve the acquisition of additional mutations.

Previous studies have reported that uterine or ovarian MLA and cervical MA showed chromosomal gains of 1q, 2, 10, 12, and 20 [40,50,52,57]. Our observation of gain of 1q, in all examined cases (13/13), is in agreement with that of our previous study, which revealed 1q gain in all except one case of uterine MLA (11/12) [40]. In addition, we previously found that the most common alterations observed in cervical MA were gains of chromosome 1q (4/4) and 10 (4/4), followed by gains of chromosome 2 (3/4), 12 (3/4), and 20 (3/4) [58]. In MLAs and MAs, da Silva et al. [52] observed frequent gains of 1q (32/36) and 10 (16/36). Interestingly, they also reported that ovarian MLAs (10/15) exhibited gain of chromosome 12 more frequently than cervical MA (2/8) or uterine MLA (2/13), while loss of chromosome 9 was more frequently identified in cervical MA (4/8) than in ovarian (1/15) or uterine MLA (1/13).

To the best of our knowledge, this study is the second to report PD-L1 expression in uterine MLA. Horn et al. [59] first reported PD-L1 negativity in four cases of uterine MLA. In line with this finding, we observed that the majority of cases (14/17) exhibited a complete lack of PD-L1 immunoreactivity in both the tumor and immune cells (CPS 0). The remaining three cases focally expressed PD-L1, with CPS < 1. In addition, our observations of retained MMR protein expression and MSS in all examined cases are consistent with our previous findings [41]. We re-examined some uterine MLA cases misinterpreted as MMRd during the initial diagnosis and found that they should have been interpreted as MMRp tumors, confirming that they were MSS. A PD-1 inhibitor, pembrolizumab, was approved for advanced, recurrent, or metastatic MSI-H/MMRd ECs as a second-line treatment. Recently, the combination of pembrolizumab with an oral multikinase inhibitor, lenvatinib, has shown remarkable results, with an objective response rate of 36% and median OS of 16.4 months for advanced non-MSI-H/MMRp ECs [24]. Although the effectiveness of lenvatinib and pembrolizumab combination therapy for uterine MLA has not been fully elucidated, there have been a few case reports documenting excellent and durable responses to this combination therapy in patients with uterine MLA [60,61,62]. Considering that the treatment of patients with advanced or recurrent uterine MLA may differ depending on MSI status, MMR IHC in uterine MLA requires careful interpretation. Repeat IHC and MSI testing may improve diagnosis of challenging cases.

The Proactive Molecular Risk Classifier for Endometrial Cancer introduced four subgroups of EC: (1) DNA polymerase epsilon, catalytic subunit (*POLE*)-mutant subgroup, harboring mutations in the exonuclease domain in exons 9–14; (2) MMRd subgroup, showing the loss of expression for one or more MMR proteins; (3) p53-abnormal subgroup, demonstrating aberrant p53 expression pattern indicating pathogenic *TP53* mutation; and (4) no specific molecular profile (NSMP) subgroup [63,64,65]. Even though the vast majority of NSMP ECs is low-grade endometrioid carcinoma, the NSMP subgroup also encompasses high-grade EC, clear cell carcinoma, undifferentiated carcinoma, carcinosarcoma, and MLA [36,40,50,51,53,57,65,66,67,68]. In this study, the majority of MLA cases harbored activating *KRAS* mutations but not *POLE*-mutant signatures, MMR deficiency, or *TP53* mutation; confirming that these cases belong to the NSMP subgroup. Since the biological behavior of uterine MLA is consistently described as aggressive [36,67,69], the inclusion of this entity in the high-risk non-endometrioid group appears to be justified. Several studies have identified that the high expression of the L1 cell adhesion molecule (L1CAM) and *CTNNB1* mutation can add significant prognostic information to the molecular classification of EC [70,71,72,73,74]. The significance of L1CAM expression or *CTNNB1* mutation in MLA patients has not been elucidated. Based on relatively poor MLA prognosis, compared to other histological types belonging to the NSMP subgroup [3,67], it is likely to exhibit L1CAM overexpression or harbor the *CTNNB1* mutation. Further studies with a larger cohort of uterine MLA patients are necessary to clarify the clinicopathological and prognostic significance of L1CAM expression and the *CTNNB1* mutation.

We found that 2 of the 17 uterine MLA cases harbor frameshift *TP53* mutations. One patient with stage IA MLA did not experience recurrence and is alive at 63.5 months postoperatively, while the other patient with stage IA disease developed recurrence at 8.0 months after surgery and died of disease at 25.1 months postoperatively. The *TP53* mutation is known to be extremely uncommon in malignant mesonephric lesions [37,52,57,75] and its clinicopathological significance in patients with uterine MLA has not yet been investigated. We recently experienced a case of dedifferentiated uterine MLA harboring the *TP53* mutation [39]. IHC revealed that the dedifferentiated component overexpressed p53, while the MLA component exhibited a wild-type p53 expression pattern. We cannot conclude the prognostic implication of the *TP53* mutation in uterine MLA because only a few cases were found. However, based on our observations that the majority of uterine MLA cases did not harbor the *TP53* mutation and that the clinical course of the two patients with early-stage, *TP53*-mutant MLA was inconsistent, we believe that there is an urgent need to determine the clinicopathological and prognostic significance of the *TP53* mutation in uterine MLA. Similar to previous results showing that the *TP53* mutation in ‘multiple-classifier’ EC cases does not significantly affect disease development or prognosis [76], we hypothesize that the *TP53* mutation occurs during late stage of MLA progression and does not affect the molecular landscape, since only two frameshift *TP53* mutations were detected in this study of uterine MLAs with pathogenic *KRAS* mutations and the two cases displayed different outcomes. Further investigations are required to investigate whether *TP53* mutation as a significant predictor for patient outcome of uterine MLA or merely a passenger event with no impact on biological behavior.

Therapeutic strategies tailored to both the genetic and epigenetic features of EC are the basis of precision medicine in gynecological oncology [77]. Aberrant expression of several cancer-related gene sets has been consistently reported to be a significant contributor for EC progression [78]. In addition to those mutational changes, epigenetic alterations, including methylation, acetylation, and phosphorylation of nuclear chromatin, play a central role in EC development and progression [78,79]. Particularly, non-coding RNAs (ncRNAs) are involved in the regulation of cellular metabolism, growth, and neoplastic transformation [78,80]. They have very little or no protein-coding capability [81,82], but their expression patterns can modulate the function of oncogenes and tumor suppressors, resulting in either promotion or suppression of tumorigenesis and progression [77]. Their regulation of gene expression can occur in different steps, at epigenetic, transcriptional, and post-transcriptional levels [78]. Accumulating evidence shows that the abnormal expression of ncRNAs is associated with the prevalence and prognosis of many different types of human cancers [82,83]. Some deregulated ncRNAs have recently been suggested as potential risk factors that can better define the biological behavior of EC and be used as prognostic markers to guide the risk stratification of EC patients. It is surprising to note that the association between ncRNA and EC has only recently been emerging in the literature, and that most of the papers regarding this association have been concentrated into the last three years [77]. The following competing endogenous RNAs seemed to be associated with poor prognosis of EC: AC074212.1, ADARB2-AS1, C2orf48, C8orf49, C10orf91, FER1L4, FP671120.4, GLIS3-AS1, HOXB-AS1, LINC00483, LINC00491, LINC01143, LINC01352, LINC01410, LINC02381, MIR503HG, PCAT1, RP11-357H14.17 and RP11 89K21.1 [77]. In contrast, LINC00237, LINC00475, LINC00958, and LNCTAM34A were reported to exhibit a favorable prognostic effect in EC patients. The expressions of these molecules were found to be deregulated in EC compared to normal endometrial tissue. Identifying deregulated microRNAs (miRs) remains an ongoing endeavor. miRs are short molecules of ncRNA that function as post-transcriptional regulators of gene expression [84]. In a meta-analysis by Delangle et al. [85], a number of significantly deregulated miRs were identified and classified as onco-miRs, suppressor miRs, and those with discordant functions. Similarly, a recent systematic review by Bloomfield et al. [86] revealed the deregulated levels of circulating miRs in the serum and plasma of EC patients. These studies suggest that adequate combinations of miR expression with conventional pathological parameters of EC may serve as prognostic markers that can help in predicting risk stratification of patients. Taken together, epigenetic modifications are gaining increasing importance for the characterization of EC. A group of molecules is emerging as identifiable risk factors that aid in establishing an accurate diagnosis and assessing clinical prognosis. Particularly, there is a significant correlation between the alterations of several ncRNAs and miRs and the clinical course of EC patients, representing the possibility of including these molecules in stratifying patients at greater risk of relapse and worse outcome [82]. A comprehensive analysis of these molecules is the way to pursue towards personalized medicine, in which each patient is characterized by a specific set of epigenetic alterations, whose targets are well defined, and for whom drawing a therapeutic strategy would yield better results [80,82].

## 5. Conclusions

We comprehensively investigated the clinicopathological and immunophenotypical features of 17 consecutive cases of uterine MLA from a single institution. We confirmed that uterine MLA is an aggressive malignancy, showing advanced stage, frequent post-operative disease recurrence, and frequent lung metastasis. Initial serosal extension and lung metastasis were independent prognostic factors for RFS prediction, while none of the clinicopathological or molecular features were significantly associated with OS of uterine MLA patients. IHC revealed that none of the cases overexpressed PD-L1 or were MMR deficient. We conducted targeted sequencing to analyze the molecular features of uterine MLA. We found that the majority of cases harbored pathogenic *KRAS* mutations. Two cases harboring the frameshift *TP53* mutation were also identified, but the clinicopathological and prognostic significance of *TP53* mutation could not be determined. The most frequent abnormalities were gains of chromosome 1q, 2, 10, and 20. Both clinicians and pathologists should be aware of these features to establish an accurate diagnosis of uterine MLA and to ensure appropriate therapeutic management of this rare entity.

## Figures and Tables

**Table 1:** Baseline clinicopathological characteristics of uterine mesonephric-like adenocarcinoma (MLA).

Case No	Age (Years)	Initial Bx Dx	Surgical Procedure	Initial Hysterectomy Dx	Initial Serosal Extension	Initial LN Metastasis	Initial Distant Metastasis	Initial Stage	Post-Operative Treatment	Post-Treatment Recurrence	Initial or Recurrent Lung Metastasis	RFS (Months)	Survival Status	OS (Months)
1	54	ADC	TH, BSO	MLA	Yes	No	No	IIIB	CCRT	Yes	Yes	1.1	Dead	28.2
2	65	MLA	TH, BSO, PLND, PALND	MLA	No	No	No	IB	RT	Yes	Yes	51.6	Alive	63.6
3	70	EC-G3	TH, BSO, PLND, PALND, PRT	MLA	No	No	No	IB	RT	No	No	66.5	Alive	66.5
4	53	ADC/DDx	TH, BSO, PLND, PALNS	MLA	No	No	No	IA	No	No	No	63.5	Alive	63.5
5	60	CS	TH, BSO, PLND, PALND	CS	No	No	No	IA	CTx	Yes	Yes	8.0	Dead	25.1
6	52	ADC	RH, BSO, PLND, PALND	EC-G1	No	No	No	IB	No	Yes	Yes	8.9	Dead	85.6
7	61	MLA	TH, BSO, PLND, PALND	MLA	No	No	No	IB	CTx	No	No	63.1	Alive	63.1
8	65	ADC/DDx	RH, RSO, PRT	MLA	Yes	No	Yes	IVB	CCRT	Yes	No	1.3	Alive	53.8
9	61	PDC	RH, BSO, PALND, COL, PRT, OMT, APP	MLA	Yes	No	Yes	IVB	CTx	Yes	Yes	9.2	Dead	32.7
10	52	EC-G2	TH, BSO, PLND, PRT	MLA	No	Yes	No	IIIC	CCRT	No	No	39.3	Alive	39.3
11	67	CS	TH, BSO, PLND, PRT	CS	No	No	No	IB	No	Yes	Yes	13.4	Alive	14.5
12	59	ADC/DDx	TH, BSO, PLND, PALNS	MLA	No	No	No	IA	No	No	No	60.0	Alive	60.0
13	75	CS	TH, BSO, PALNS	CS	No	No	No	IB	No	Yes	Yes	15.6	Dead	17.3
14	55	EC-G2	TH, BSO, OMT	EC-G2	No	No	Yes	IVB	CCRT	Yes	Yes	15.7	Alive	94.6
15	57	ADC/DDx	RH, BSO, PLND, PALND, APP	CS	Yes	Yes	No	IIIC	CCRT	Yes	Yes	12.1	Alive	65.4
16	52	NA	TH, BSO, PLND, PALND, OMT, APP	MLA	No	No	No	IIIB	CCRT	No	No	45.1	Alive	45.1
17	60	EC-G2	TH, BSO, PLND, PALND	EC-G3	No	Yes	No	IIIB	CCRT	Yes	No	16.1	Dead	35.3

Abbreviations: ADC—Adenocarcinoma; APP—appendectomy; BSO—bilateral salpingo-oophorectomy; Bx—biopsy; CCRT—concurrent chemoradiation therapy; COL—colectomy; CS—carcinosarcoma; CTx—chemotherapy; Dx—diagnosis; DDx—differential diagnosis; EC-G1—endometrioid carcinoma, grade 1; EC-G2—endometrioid carcinoma, grade 2; EC-G3—endometrioid carcinoma, grade 3; LN—lymph node; NA—not applicable; OMT—omentectomy; PALND—para-aortic lymph node dissection; PALNS—para-aortic lymph node sampling; PLND—pelvic lymph node dissection; PDC—poorly differentiated carcinoma; PRT—peritonectomy; OMT—omentectomy; OS—overall survival; RT—radiation therapy; PRT—peritonectomy; RH—radical hysterectomy; RFS—recurrence-free survival; RSO—right salpingo-oophorectomy; TH—total hysterectomy.

**Table 2:** Single nucleotide variations (SNVs) and copy number variations (CNVs) detected using targeted sequencing.

Case No	Tumor Location	SNV					CNV	
		Gene	Sequence Change	Amino Acid Change	VAF (%)	Type	Gene Gain	Gene Loss
1	Uterus	*KRAS*	c.34G>T	p.G12C	40.5	MS	*NTRK1*, *ARID1B*, *JAK2*, *GNAQ*, *RET*, *HNF1A*, *SRC*, *GNAS*	*JAK1*, *CTNNB1*, *PIK3CA*, *ROS1*
2	Uterus	*KRAS*	c.34G>T	p.G12C	84.7	MS	*NTRK1*, *DDR2*, *ALK*, *ERBB4*, *IDH1*, *RET*, *FGFR2*, *MDM2*, *SRC*	None
		*PTEN*	c.804C>A	p.D268E	20.6	MS		
		*NF1*	c.4942A>G	p.T1648A	42.2	MS		
		*ARID1A*	c.863_875del	p.Q288Pfs*71	60.3	FS		
		*GNAQ*	c.303C>A	p.Y101*	7.9	NS		
3	Uterus	*KRAS*	c.34G>T	p.G12C	62.2	MS	*NTRK1*, *DDR2*, *ALK*, *ERBB4*, *FGFR2*, *RET*, *SRC*, *TOP1*, *GNAS*	None
4	Uterus	*KRAS*	c.35G>T	p.G12V	71.8	MS	*NTRK1*, *HNF1A*, *CSF1R*, *CDKN2A*, *GNAQ*, *PTCH1*, *JAK3*	None
		*TP53*	c.210_211insG	p.P71fs*78	47.6	FS		
		*RB1*	c.1861C>A	p.R621S	52.5	MS		
		*GNAQ*	c.303C>A	p.Y101*	6.6	NS		
5	Uterus	*KRAS*	c.37G>T	p.G13C	78.4	MS	*NTRK1*, *DDR2*, *ALK*, *ERBB4*, *CSF1R*, *CDKN2A*, *GNAQ*, *FGFR2*, *TOP1*, *GNAS*	None
		*TP53*	c.216delC	p.V73fs	72.3	FS		
6	Uterus	*KRAS*	c.35G>A	p.G12D	97.4	MS	NA	NA
		*BCR*	c.3316G>A	p.D1106N	9.8	MS		
7	Uterus	*KRAS*	c.35G>T	p.G12V	39.9	MS	NA	NA
		*CTNNB1*	c.134C>T	p.S45F	4.1	MS		
		*IDH1*	c.623A>G	p.Y208C	53	MS		
		*GNAQ*	c.303C>A	p.Y101*	7.9	NS		
8	Uterus	*KRAS*	c.53G>C	p.G12A	65.9	MS	NA	NA
		*RB1*	c.1666C>T	p.R556*	28.1	NS		
		*BCR*	c.3316G>A	p.D1106N	10.6	MS		
9	Uterus	*KRAS*	c.35G>T	p.G12V	58	MS	NA	NA
10	Uterus	*KRAS*	c.35G>A	p.G12D	61.2	MS	NA	NA
		*PIK3CA*	c.1633G>A	p.E545K	41.3	MS		
		*CDH1*	c.2638G>A	p.E880K	6.8	MS		
11	Uterus	*PIK3CA*	c.317G>T	p.G106V	49.2	MS	NA	NA
		*NF1*	c.2991-1G>C	NA	21.1	SS		
12	Uterus	*PTEN*	c.804C>A	p.D268E	17.9	MS	NA	NA
		*PIK3R1*	c.1690A>G	p.N564D	6.4	MS		
13	Uterus	*PTEN*	c.70G>C	p.D24H	95.5	MS	NA	NA
		*PIK3CA*	c.2740G>A	p.G914R	94.8	MS		
14-1	Uterus	*KRAS*	c.35G>T	p.G12V	56.56	MS	*NTRK1*, *DNMT3A*, *RET*, *FGFR2*, *RAB35*, *POLE*	*JAK1*
14-2	Lung	*KRAS*	c.35G>T	p.G12V	39.31	MS	*NTRK1*, *DDR2*, *ABL2*, *FGFR2*, *ERBB3*, *POLE*	None
15-1	Uterus	*KRAS*	c.35G>A	p.G12D	83.25	MS	*NTRK1*, *MDM4*, *AKT3*, *ALK*, *DNMT3A*, *MYCN*, *ERBB4*, *RET*, *FGFR2*	None
15-2	LN	*KRAS*	c.35G>A	p.G12D	45.73	MS	*ALK*, *DNMT3A*, *MAP3K4*, *FGFR2*	None
16-1	Uterus	*KRAS*	c.35G>A	p.G12D	12.52	MS	*DDR2*, *SMAD4*	*JAK2*
16-2	Ovary	*KRAS*	c.35G>A	p.G12D	34.27	MS	*NTRK1*, *DDR2*, *ABL2*, *DNMT3A*, *FGFR2*	None
17-1	Uterus	*PTEN*	c.892C>T	p.Q298*	32.56	NS	*DDR2*, *ABL2*, *MDM4*	None
17-2	LN	*PTEN*	c.892C>T	p.Q298*	28.09	NS	*NTRK1*, *DDR2*	None

Abbreviations: *ABL2*—Abelson murine leukemia viral oncogene homolog 2; *AKT3*—Akt murine thymoma viral oncogene homolog 3; *ALK*—anaplastic lymphoma receptor tyrosine kinase; *ARID1A*—AT-rich interactive domain 1A; *ARID1B*—AT-rich interactive domain 1B; *BCR*—breakpoint cluster region; *CDH1*—cadherin 1; *CDKN2A*—cyclin-dependent kinase inhibitor 2A; *CSF1R*—colony-stimulating factor 1 receptor; *CTNNB1*—catenin beta 1; *DDR2*—discoidin domain receptor tyrosine kinase 2; del—deletion; *DNMT3A*—DNA methyltransferase 3 alpha; *ERBB3*—erb-B2 receptor tyrosine kinase 3; *ERBB4*—erb-B2 receptor tyrosine kinase 4; *FGFR2*—fibroblast growth factor receptor 2; FS—frameshift; *GNAQ*—guanine nucleotide binding protein subunit alpha Q; *GNAS*—guanine nucleotide binding protein, alpha stimulating activity polypeptide 1; *HNF1A*—hepatocyte nuclear factor 1-alpha; *IDH1*—isocitrate dehydrogenase 1; ins—insertion; *JAK1*—Janus kinase 1; *JAK2*—Janus kinase 2; *JAK3*—Janus kinase 3; *KRAS*—Kirsten rat sarcoma viral oncogene homolog; LN—lymph node; *MAP3K4*—mitogen-activated protein kinase 4; *MDM2*—mouse double minute 2 proto-oncogene; *MDM4*—mouse double minute 4 regulator of p53; MS—missense; *MYCN*—myelocytomatosis viral oncogene neuroblastoma derived homolog; NA—not applicable; *NF1*—neurofibromin 1; NS—nonsense; *NTRK1*—neurotrophic receptor tyrosine kinase 1; *PIK3CA*—phosphatidylinositol-4,5-bisphosphate 3-kinase catalytic subunit alpha; *PIK3R1*—phosphoinositide-3-kinase regulatory subunit 1; *POLE*—DNA polymerase epsilon catalytic subunit; *PTCH1*—patched 1; *PTEN*—phosphatase and tensin homolog deleted on chromosome 10; *RAB35*—Ras-related protein Rab-35; *RB1*—retinoblastoma 1; *RET*—rearranged during transfection; *ROS1*—repressor of silencing 1; *SMAD4*—mothers against decapentaplegic homolog 4; *SRC*—v-src avian sarcoma (Schmidt–Ruppin A-2) viral oncogene homolog; SS—splice site mutation; *TOP1*—DNA topoisomerase I; *TP53*—tumor protein p53; VAF—variant allele frequency.

**Table 3:** Results of immunohistochemical staining (IHC) for programmed cell death-ligand 1 (PD-L1) and mismatch repair (MMR) proteins, microsatellite instability (MSI) testing, and tumor mutational burden (TMB) quantification.

Case No	MSI Status	PD-L1 22C3 Pharmdx CPS	MMR IHC	TMB (per Mb)
1	MSS	0	MMR-proficient	3.28
2	MSS	0	MMR-proficient	3.99
3	MSS	0	MMR-proficient	4.05
4	MSS	0	MMR-proficient	4.15
5	MSS	0	MMR-proficient	4.38
6	MSS	0	MMR-proficient	2.70
7	NA	0	MMR-proficient	NA
8	NA	0	MMR-proficient	NA
9	MSS	0	MMR-proficient	3.44
10	MSS	0	MMR-proficient	3.47
11	MSS	0.5	MMR-proficient	3.51
12	MSS	0.5	MMR-proficient	3.54
13	MSS	0	MMR-proficient	3.57
14	MSS	0	MMR-proficient	3.67
15	MSS	0.1	MMR-proficient	4.15
16	MSS	0	MMR-proficient	4.72
17	MSS	0	MMR-proficient	3.38

Abbreviations: CPS—Combined proportion score; Mb—mega base; MSS—microsatellite-stable; NA—not applicable.

**Table 4:** Clinicopathological significance of recurrent pathogenic mutations.

Characteristic		*KRAS*			*PTEN*			*PIK3CA*			*PTEN* or *PIK3CA*		
		Wild Type	Mutant	*p*-Value	Wild Type	Mutant	*p*-Value	Wild Type	Mutant	*p*-Value	Wild Type	Mutant	*p*-Value
Age (years)	<60	1 (12.5)	7 (87.5)	0.66	7 (87.5)	1 (12.5)	0.66	7 (87.5)	1 (12.5)	1.00	6 (75.0)	2 (25.0)	0.74
	≥60	3 (33.3)	6 (66.7)		6 (66.7)	3 (33.3)		7 (77.8)	2 (22.2)		5 (55.6)	4 (44.4)	
Initial serosal extension	No	4 (30.8)	9 (69.2)	0.55	9 (69.2)	4 (30.8)	0.55	10 (76.9)	3 (23.1)	0.76	7 (53.8)	6 (46.2)	0.28
	Yes	0 (0.0)	4 (100.0)		4 (100.0)	0 (0.0)		4 (100.0)	0 (0.0)		4 (100.0)	0 (0.0)	
Initial lymph node metastasis	No	4 (26.7)	11 (73.3)	1.00	11 (73.3)	4 (26.7)	1.00	13 (86.7)	2 (13.3)	0.77	10 (66.7)	5 (33.3)	1.00
	Yes	0 (0.0)	2 (100.0)		2 (100.0)	0 (0.0)		1 (50.0)	1 (50.0)		1 (50.0)	1 (50.0)	
Initial distant metastasis	No	4 (28.6)	10 (71.4)	0.76	10 (71.4)	4 (28.6)	0.76	11 (78.6)	3 (21.4)	0.96	8 (57.1)	6 (42.9)	0.46
	Yes	0 (0.0)	3 (100.0)		3 (100.0)	0 (0.0)		3 (100.0)	0 (0.0)		3 (100.0)	0 (0.0)	
Initial or recurrent lung metastasis	No	2 (25.0)	6 (75.0)	1.00	6 (75.0)	2 (25.0)	1.00	7 (87.5)	1 (12.5)	1.00	5 (62.5)	3 (37.5)	1.00
	Yes	2 (22.2)	7 (77.8)		7 (77.8)	2 (22.2)		7 (77.8)	2 (22.2)		6 (66.7)	3 (33.3)	
Initial stage	I	3 (33.3)	6 (66.7)	0.49	6 (66.7)	3 (33.3)	0.49	7 (77.8)	2 (22.2)	0.67	5 (55.6)	4 (44.4)	0.37
	II	0 (0.0)	0 (0.0)		0 (0.0)	0 (0.0)		0 (0.0)	0 (0.0)		0 (0.0)	0 (0.0)	
	III	1 (20.0)	4 (80.0)		4 (80.0)	1 (20.0)		4 (80.0)	1 (20.0)		3 (60.0)	2 (40.0)	
	IV	0 (0.0)	3 (100.0)		3 (100.0)	0 (0.0)		3 (100.0)	0 (0.0)		3 (100.0)	0 (0.0)	
Post-treatment recurrence	No	1 (16.7)	5 (83.3)	1.00	5 (83.3)	1 (16.7)	1.00	5 (83.3)	1 (16.7)	1.00	4 (66.7)	2 (33.3)	1.00
	Yes	3 (27.3)	8 (72.7)		8 (72.7)	3 (27.3)		9 (81.8)	2 (18.2)		7 (63.6)	4 (36.4)	

Abbreviations: *KRAS*—Kirsten rat sarcoma viral oncogene homolog; LN—lymph node; *PIK3CA*—phosphatidylinositol-4,5-bisphosphate 3-kinase catalytic subunit alpha; *PTEN*—phosphatase and tensin homolog deleted on chromosome 10.

**Table 5:** Clinicopathological significance of recurrent pathogenic mutations.

Characteristic		Recurrence-Free Survival			Overall Survival		
		Univariate	Multivariate		Univariate	Multivariate	
		*p*-Value	*p*-Value	HR (95% CI)	*p*-Value	*p*-Value	HR (95% CI)
Initial serosal extension	Present	<0.001 *	0.037 *	6.30 (1.12–35.45)	0.40	NA	NA
	Absent						
Initial lymph node metastasis	Present	0.80	NA	NA	0.36	NA	NA
	Absent						
Initial distant metastasis	Present	0.07	NA	NA	0.63	NA	NA
	Absent						
Initial or recurrent lung metastasis	Present	0.002 *	0.02 *	7.31 (1.38–38.75)	0.09	NA	NA
	Absent						
Initial stage	III−IV	0.25	NA	NA	0.91	NA	NA
	I−II						
*KRAS* mutation	Present	0.99	NA	NA	0.13	NA	NA
	Absent						
*PTEN* mutation	Present	0.72	NA	NA	0.36	NA	NA
	Absent						
*PIK3CA* mutation	Present	0.98	NA	NA	0.35	NA	NA
	Absent						
*PTEN* or *PIK3CA* mutation	Present	0.72	NA	NA	0.63	NA	NA
	Absent						

Abbreviations: CI—Confidence interval; HR—hazard ratio; *KRAS*—Kirsten rat sarcoma viral oncogene homolog; LN—lymph node; *PIK3CA*—phosphatidylinositol-4,5-bisphosphate 3-kinase catalytic subunit alpha; *PTEN*—phosphatase and tensin homolog deleted on chromosome 10. * Statistically significant.

## Data Availability

Not applicable.
